# Characterization of blood biochemical markers during aging in the Grey Mouse Lemur (*Microcebus murinus*): impact of gender and season

**DOI:** 10.1186/1746-6148-8-211

**Published:** 2012-11-06

**Authors:** Julia Marchal, Olène Dorieux, Laurine Haro, Fabienne Aujard, Martine Perret

**Affiliations:** 1 Mécanismes Adaptatifs et Evolution, UMR 7179 Centre National de la Recherche Scientifique, Muséum National d'Histoire Naturelle, Equipe Mécanismes Adaptatifs et Evolution, 1 Avenue du Petit Château, Brunoy 91800, France

**Keywords:** Aging, Blood biochemical markers, Grey Mouse Lemur, *Microcebus murinus*, Seasonality

## Abstract

**Background:**

Hematologic and biochemical data are needed to characterize the health status of animal populations over time to determine the habitat quality and captivity conditions. Blood components and the chemical entities that they transport change predominantly with sex and age. The aim of this study was to utilize blood chemistry monitoring to establish the reference levels in a small prosimian primate, the Grey Mouse Lemur (*Microcebus murinus*).

**Method:**

In the captive colony, mouse lemurs may live 10–12 years, and three age groups for both males and females were studied: young (1–3 years), middle-aged (4–5 years) and old (6–10 years). Blood biochemical markers were measured using the VetScan Comprehensive Diagnostic Profile. Because many life history traits of this primate are highly dependent on the photoperiod (body mass and reproduction), the effect of season was also assessed.

**Results:**

The main effect of age was observed in blood markers of renal functions such as creatinine, which was higher among females. Additionally, blood urea nitrogen significantly increased with age and is potentially linked to chronic renal insufficiency, which has been described in captive mouse lemurs. The results demonstrated significant effects related to season, especially in blood protein levels and glucose rates; these effects were observed regardless of gender or age and were likely due to seasonal variations in food intake, which is very marked in this species.

**Conclusion:**

These results were highly similar with those obtained in other primate species and can serve as references for future research of the Grey Mouse Lemur.

## Background

Both hematological and biochemical data are needed to characterize the health status of animal populations over time and to determine the habitat or captive habitat quality. Such information may be useful when screening for diseases, infections or routine check-ups in captive populations.

Numerous studies have demonstrated the importance of regular monitoring of blood biochemical markers in different contexts in various animal species (turtles [1], rattlesnakes [2], squirrels [3] and red howler monkeys [4]). Blood or serum biochemistry is effective for detecting stress induced by capture, captivity and/or research procedures. For example, the quantified haptoglobin concentrations are good indicators for controlling the stress of capture and retention in the Steller Sea Lion and to detect potential unknown stressors on free-ranging animals [5]. Recently, blood biochemical markers were used to highlight the effects of a short-term effort on dugongs [6]. Such markers have also been used to quantify and to compare blood acid–base disturbances associated with capture and to investigate how interspecific differences in the physiological stress response could be related to life history ecology and phylogeny [7]. Likewise, comparisons of blood biochemistry levels between species could be useful to prevent diseases or pre-disease states, such as kidney dysfunction in chimpanzees and humans [8] or rhabdomyolysis in marine mammals [6]. Finally, blood chemistry profiles are used as biomarkers for disease evolution, including atherosclerosis in rabbits [9] or organohalogen contamination in raptor nestlings [10].

Blood components change based on several factors, particularly sex and age, but few studies follow-up these biochemical markers long term. There have been only a few studies on animal development, especially in primate species. A chimpanzee report described the utility of following hematological and biochemical markers in cross-sectional and longitudinal analyses [11]. Blood biochemical parameters are subject to changes with increasing age in many animal species [12,13]. In humans, reference values of several blood components in young, middle-aged, old and centenarian individuals have been established [14,15].

From long-term studies, blood biomarkers may be used to define the biological age of an individual by comparing its various biochemical parameters. For some species, including humans, studies have reported standard values (primates [16]; humans [14,17,18]). However, standard values for exotic species or new primate experimental models, such as the Grey Mouse Lemur, are not available.

The main objective of this study was to utilize cross-sectional blood chemistry monitoring to establish the reference values of blood biochemical parameters in the Grey Mouse Lemur (*Microcebus murinus*), a nocturnal prosimian primate used as a model for aging research [19]. This species may live in captivity for 10–12 years and exhibits photoperiod-dependent behavioral and physiological changes. Mouse lemurs have an exceptional lifespan compared to other mammalian species of similar size, such as rodents. Mouse lemurs may live up to two or three times longer than mammals of equivalent body mass [20], which is an advantage for research on key mechanisms underlying aging. Longitudinal studies are currently underway in the breeding colony of Brunoy [19], but no study has previously reported the effects of aging on blood biochemical markers in this species. Data were collected over a three-year period on 78 mouse lemurs, ranging in age from 1 to 10 years; this cohort represents approximately 15% of the Brunoy breeding colony. The potential effects of sex, season and age were analyzed. Our study may provide a reference for basic diagnostic and clinical care of mouse lemurs in captivity or in the field.

## Results

### Body mass

**Figure 1:**
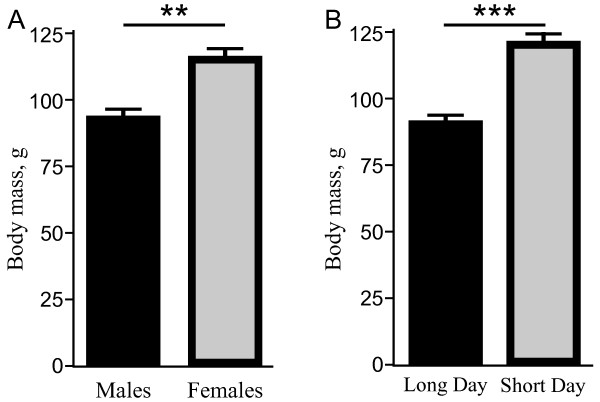
**Means and SEM of mouse lemur body mass (g) was significantly different between (A) sexes (males and females) and (B) seasons (long-day season; short-day season).** Statistical significance was considered when p<0.01 (**) and p<0.001 (***).

The body mass of mouse lemurs varied significantly between the two sex groups and between the seasons, and we observed important general effects of sex (dF_1/67_, F=7.094, p<0.01) and season (dF_1/67_, F=20.45, p<0.001). Sex-specific and seasonal differences were independent of age (dF_2/67_, F=0.284, p=0.75). Regardless of age, male mouse lemurs had a 20% lower body mass than females, and animals in the long-day (LD) season had a 22% lower body mass than animals in the short-day (SD) season (Figure 1).

The blood biochemical markers values obtained from the Grey Mouse Lemurs were represented by gender in Tables 1 and 2, according to the short-day season and to the long-day season respectively.

**Table 1:** Blood biochemical markers during the short-day season

	**Short-day season**					
	**Male**			**Female**		
**Parameter (units)**	**Median**	**25-75% quartiles**	**Range (min-max)**	**Median**	**25-75% quartiles**	**Range (min-max)**
**Na (mmol/L)**	**141**	**138 - 143**	**138 - 147**	**146**	**141 - 148**	**48 - 152**
**K (mmol/L)**	**6.45**	**6.1 - 7.2**	**5.9 - 7.8**	**7**	**6 - 7.7**	**2.4 - 8.5**
**Ca (mg/dL)**	**9.9**	**9.5 - 10.3**	**9.4 - 10.8**	**10.3**	**10.1 - 10.7**	**4.1 - 11**
**PHOSP (mg/dL)**	**5.1**	**4.3 - 5.7**	**4.3 - 8.1**	**5.4**	**4.6 - 6**	**2.6 - 13**
**GLU (mg/dL)**	**100**	**81 - 107**	**67 - 169**	**101**	**75 - 117**	**51 - 171**
**ALP (U/L)**	**52**	**45 - 66**	**26 - 74**	**49**	**37 - 51**	**16 - 202**
**ALT (U/L)**	**200**	**158 - 283**	**83 - 591**	**179**	**129 - 242**	**71 - 495**
**AMY (U/L)**	**226**	**43 - 286**	**38 - 423**	**161**	**59 - 227**	**32 - 356**
**CRE (mg/dL)**	**0.2**	**0.1 - 0.2**	**0 - 0.6**	**0.3**	**0.2 - 0.5**	**0.1 - 3.2**
**BUN (mg/dL)**	**15**	**13 - 23**	**12 - 34**	**18**	**14 - 24**	**5 - 77**
**TP (g/dL)**	**9.8**	**9.1 - 10.1**	**9.1 - 11**	**11.3**	**10.1 - 11.9**	**3.5 - 13.7**
**ALB (g/dL)**	**6.2**	**5.7 - 6.5**	**4.8 - 6.5**	**5.6**	**5 - 6.5**	**1.6 - 6.5**
**TBIL (mg/dL)**	**0.4**	**0.4 - 0.4**	**0.3 - 0.5**	**0.4**	**0.3 - 0.4**	**0 - 0.5**
**GLOB (g/dL)**	**3.7**	**0 - 4.2**	**0 - 4.7**	**4.5**	**0 - 5.4**	**0 - 7.8**

Median, 25%-75% quartiles and range (minimum-maximum) of blood biochemical markers during the short-day season in male and female mouse lemurs.

**Table 2:** Blood biochemical markers during the long-day season

	**Long-day season**					
	**Male**			**Female**		
**Parameter (units)**	**Median**	**25-75% quartiles**	**Range (min-max)**	**Median**	**25-75% quartiles**	**Range (min-max)**
**Na (mmol/L)**	**147**	**143 - 149**	**100 - 151**	**148**	**146 - 149**	**116 - 151**
**K (mmol/L)**	**7.25**	**6.4 - 7.9**	**2.3 - 8.5**	**7.8**	**7.1 - 7.9**	**5.8 - 8.5**
**Ca (mg/dL)**	**10.3**	**9.8 - 10.6**	**4.2 - 12.2**	**10.3**	**9.6 - 10.5**	**8.1 - 10.9**
**PHOSP (mg/dL)**	**6.2**	**5 - 8.1**	**2.6 - 12.6**	**6.9**	**5.9 - 8.1**	**5.1 - 12.7**
**GLU (mg/dL)**	**116**	**89 - 144**	**51 - 226**	**114**	**108 - 130**	**75 - 192**
**ALP (U/L)**	**58**	**47 - 80**	**26 - 87**	**58**	**49 - 64**	**28 - 144**
**ALT (U/L)**	**204**	**143 - 262**	**75 - 377**	**218**	**166 - 368**	**116 - 749**
**AMY (U/L)**	**165**	**99 - 249**	**33 - 1362**	**175**	**125 - 242**	**33 - 680**
**CRE (mg/dL)**	**0.2**	**0.2 - 0.3**	**0.2 - 0.6**	**0.3**	**0.3 - 0.4**	**0.2 - 1.1**
**BUN (mg/dL)**	**17.5**	**13 - 21**	**6 - 33**	**17.5**	**12 - 26**	**8 - 103**
**TP (g/dL)**	**8.2**	**7.8 - 8.9**	**2.9 - 9.8**	**8.6**	**8 - 8.8**	**6 - 9.3**
**ALB (g/dL)**	**4.85**	**4.4 - 5.2**	**1.5 - 5.6**	**5.1**	**4.8 - 5.4**	**3.4 - 5.6**
**TBIL (mg/dL)**	**0.4**	**0.3 - 0.4**	**0.2 - 0.5**	**0.3**	**0.3 - 0.4**	**0.3 - 0.5**
**GLOB (g/dL)**	**3.2**	**3 - 4.1**	**1.4 - 5.1**	**3.25**	**3 - 4**	**2.3 - 5.4**

Median, 25%-75% quartiles and range (minimum-maximum) of blood biochemical markers during the long-day season in male and female mouse lemurs.

### Electrolytes

#### *Sodium (Na)*

Blood Na concentrations averaged 145.5 mmol/L, which was independent of sex (dF_1/66_, F=0.006, p=0.94), season (dF_1/66_, F=0.649, p=0.42) and age (dF_2/66_, F=1.263, p=0.29).

#### *Potassium (K)*

Blood K concentrations averaged 7 mmol/L, which was not significantly different for different sexes (dF_1/54_, F=0.462, p=0.5), seasons (dF_1/54_, F=0.65, p=0.42) or ages (dF_2/54_, F=0.938, p=0.39).

#### *Calcium (Ca)*

Blood Ca levels averaged 10 mg/dL, and no differences were observed between sexes (dF_1/67_, F=0.447, p=0.51), seasons (dF_1/67_, F=0.06, p=0.81) and ages (dF_2/67_, F=0.676, p=0.51).

#### *Phosphorus (PHOSP)*

**Figure 2:**
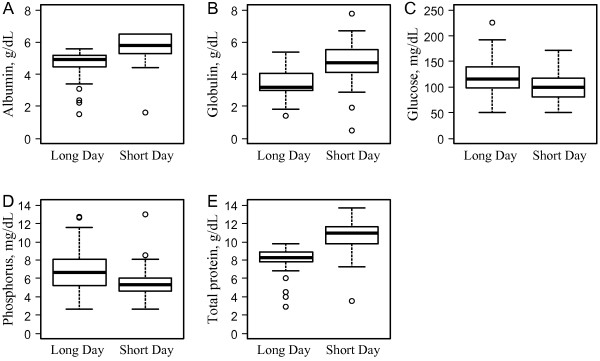
Medians and the 25% and 75% quartiles of blood biochemical markers that were significantly different between seasons (long-day season; short-day season) in Grey Mouse Lemurs.

By contrast, PHOSP concentrations were significantly higher in the LD season than values observed in the SD season (dF_1/67_, F=7.501, p<0.01); this difference was independent of sex (dF_1/67_, F=1.71, p=0.19) or age (dF_2/67_, F=0.184, p=0.83) (Figure 2).

### Glycemia

#### *Glucose (GLU)*

A significant seasonal effect was observed for blood glucose concentrations (dF_1/67_, F=4.351, p=0.04); higher GLU values were observed in the LD season regardless of gender (dF_1/67_, F=0.04, p=0.84) or age (dF_2/67_, F=0.811, p=0.45) (Figure 2).

### Enzymatic activity

#### *Alkaline phosphatase activity (ALP)*

Measurements of ALP averaged 55 U/L, and no significant differences were observed for the various sexes, seasons or ages (dF_1/67_, F=0.183, p=0.67; dF_1/67_, F=1.54, p=0.22; and dF_2/67_, F=0.996, p=0.37, respectively).

#### *Alanine aminotransferase activity (ALT)*

ALT values averaged 200 U/L, and the values did not significantly vary between the sexes, seasons or ages (dF_1/67_, F=0.029, p=0.86; dF_1/67_, F=1.103, p=0.29; and dF_2/67_, F=0.161, p=0.85, respectively).

#### *Amylase (AMY)*

Blood amylase values were highly variable among animals (from 32 to 1362 U/L), and no significant effect of sex, season or age was observed (dF_1/66_, F=0.525, p=0.47; dF_1/66_, F=0.445, p=0.5; and dF_2/66_, F=0.059, p=0.94, respectively).

### Renal function markers

#### *Creatinine (CRE)*

**Figure 3:**
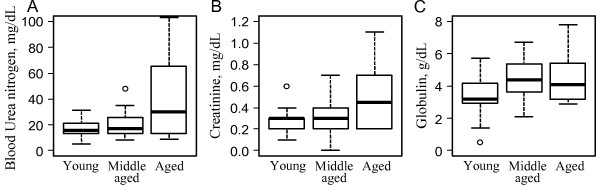
Medians and 25% and 75% quartiles of blood biochemical markers that were significantly different between age groups (young animals: 1 to 3 years old; middle-aged animals: 4 to 5 years old; and old animals: 6 to 10 years old).

Creatinine levels (average 0.25 mg/dL) varied according to age (dF_2/64_, F=5.99, p<0.01) (Figure 3) and between sexes (dF_1/64_, F=4.349, p=0.04). Higher CRE values were observed in females compared to males, which was independent of season (dF_1/64_, F=0.011, p=0.91). While middle-aged animals had no difference in CRE levels compared to young animals (p=0.80), old animals had significantly higher levels of CRE when compared to young animals levels (p<0.01).

#### *Blood urea nitrogen (BUN)*

Blood urea concentrations averaged 17 mg/dL, and a significant effect of age (dF_2/67_, F=6.95, p<0.01) (Figure 3) was observed, which was independent of sex (dF_1/67_, F=0.032, p=0.86) and season (dF_1/67_, F=0.291, p=0.59). Old animals had higher BUN levels when compared to young animals (p<0.001), but there was no difference between BUN levels of middle-aged and young animals (p=0.19) (Figure 3).

### Proteins levels

#### *Total protein (TP)*

In mouse lemurs, TP values varied from 3 to 12 g/dL and were significantly higher during the SD season compared to the LD season (dF_1/67_, F=13.43, p<0.001). No differences were observed between the sexes (dF_1/67_, F=1.33, p=0.25) or age groups (dF_2/67_, F=0.943, p=0.39) (Figure 2).

#### *Albumin (ALB)*

Like TP levels, ALB values were significantly different between the seasons, and higher levels were observed during the SD season (dF_1/63_, F=8.22, p<0.01). No differences were observed between the sexes (dF_1/63_, F=0.012, p=0.91) or age groups (dF_2/63_, F=0.467, p=0.63) (Figure 2).

#### *Total bilirubin (TBIL)*

Bilirubin values did not significantly differ between seasons (dF_1/61_, F=1.613, p=0.21) or age groups (dF_2/61_, F=1.368, p=0.26), though TBIL levels tended to be lower in females (dF_1/61_, F=3.809, p=0.055).

#### *Globulin concentration (GLOB)*

GLOB levels averaged 3.6 g/dL but were significantly impacted by season; higher GLOB levels were detected in the LD season compared to the SD season (dF_1/52_, F=12.35, p<0.001) (Figure 2). GLOB levels were also modified by age (dF_2/52_, F=4.56, p=0.01); both seasonal and age effects were independent of sex (dF_1/52_, F=2.92, p=0.09). Middle-aged animals had higher levels of GLOB compared to young animals (p<0.01), and old animals had significantly higher levels of GLOB when compared to young animals (p=0.02) (Figure 3).

### Relationship between body mass and biochemical markers

Due to the high seasonal changes in physiological functions, potential relationships between blood markers and body mass have been evaluated (Table 3).

**Table 3:** Relationship between blood biochemical parameters and body mass

**Dependent variables**	**Independent variables**	**LD season**	**SD season**
**ALP (U/L)**	**Body mass (g)**	n=39, r=0.10	n=37, r=0.32 *
**BUN (mg/dL)**		n=39, r=0.14	n=37, r=0.41 **
**GLU (mg/dL)**		n=39, r=0.16	n=37, r=0.43 **
**PHOSP (mg/dL)**		n=39, r=0.16	n=37, r=0.37 *

Sample sizes for each photoperiod: long-day (LD) season and short-day (SD) season and the Pearson correlation coefficient r are given for each regression, as dependent and independent variables. Values were statistically significant if p<0.05 (*). A p<0.01 was indicated by two asterisks (**).

ALP levels and body mass were significantly correlated in the SD season (r=0.32, p=0.03). However, this relationship was not observed in the LD season (r=0.10, p =0.46). Similarly, BUN levels and body mass were highly correlated in the SD season (r=0.41, p<0.01) but not in the LD season (r=0.14, p= 0.63). In addition, during the SD season, GLU levels exhibited a strong positive correlation with body mass (r=0.43, p<0.01), which was not true for GLU levels in the LD photoperiod (r=0.16, p=0.77). Finally, there was a significant relationship between PHOSP measures and body mass in the SD season (r=0.37, p=0.01), while no relation was observed during the LD season (r=0.16, p=0.83).

### Relationship between chronological age and biochemical markers

**Figure 4:**
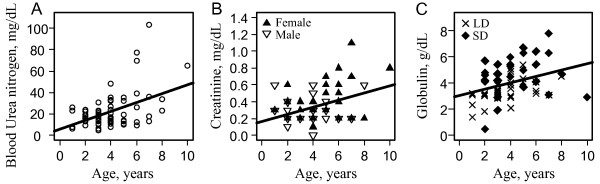
**Statistical analyses were performed utilizing a Pearson correlation.** (**A**) Simple linear regression between blood urea nitrogen levels and age (n=77, r=0.46, p<0.001). (**B**) Simple linear regression between creatinine levels and age (n=75, r=0.28, p<0.01). (**C**) Simple linear regression between total blood globulin concentration and age (n=73, r=0.20, p<0.05), LD: long-day season; SD: short-day season.

We tested the relationship between blood markers and animal age in years. Chronological age was considered to be the independent variable. Blood urea nitrogen, creatinine and globulin levels increased sharply as age increased; this relationship was illustrated by the significant correlation between chronological age and these parameters (BUN: r=0.46, p<0.001; CRE: r=0.28, p<0.01; and GLOB: r=0.20, p=0.05) (Figure 4).

## Discussion

The potential effects of gender, season and age on blood parameters were characterized for the first time in mouse lemurs through a cross-sectional study.

A comparison of mouse lemur biochemical values to “normal” biochemical blood parameters recorded in other mammals and primate species revealed several similarities and discrepancies depending on the parameter tested. Compared to ranges described for mice and rats (VetScan references for small animals), mouse lemurs had similar levels of albumin, alkaline phosphatase activity, total bilirubin and calcium levels, but mouse lemurs exhibited higher levels of alanine aminotransferase activity, blood urea nitrogen, creatinine and total protein. Compared to other non-human primate species, mouse lemurs had lower albumin and total protein levels than squirrel monkeys (*Saimiri sciureus*), macaque rhesus (*Macaca mulatta)* and cynomolgus monkeys (*Macaca fascicularis*)), while mouse lemurs exhibited higher blood urea nitrogen values relative to these primates (Fiocruz Primate Center [16]). We also compared the mouse lemur parameters with values observed in chimpanzees [11]. Creatinine, calcium and total bilirubin did not differ between chimpanzees and mouse lemurs. However, levels of glucose, blood urea nitrogen, phosphorus, potassium, total protein, albumin and globulin were higher in mouse lemurs than in chimpanzees. The most important difference in these species was the alanine aminotransferase activity; this activity was approximately five times higher in mouse lemurs compared to other primate species. The liver is the site of many biochemical and enzymatic activities, and it represents 2% of the total body weight in humans and approximately 8% of the total body weight of the mouse lemur (unpublished data). This discrepancy in the liver weight/body weight ratio may explain the high levels of alanine aminotransferase in the mouse lemur. In addition, stress is a key factor causing variations in blood parameter measurements; thus, anesthesia is often used prior to sample collection. In this study, however, anesthesia was not used. Breeding animals are accustomed to repeated handling, and not using anesthesia prevented disruptions in blood metabolite concentrations due to active anesthetics.

### Sex differences

In mouse lemurs, no sex-specific differences were observed in blood biochemical values except for creatinine levels, which were higher in females. In many species, serum creatinine levels have been shown to be generally higher in males (non-human primates [21,22]; apes [11]; and humans [23]). This gender difference is due to higher muscle mass and increased turnover in males compared to females [24]. It is noteworthy that female *M. murinus* are heavier than males, both in the wild [25] and in captivity, regardless of the season. However, despite a significant 20% higher body mass in females, no relationship between body mass and creatinine levels was found. This result suggests that either female body mass is not related to increased free fat mass or that other parameters are involved.

### Seasonal differences

Almost half of the 14 parameters studied were impacted by seasonality and could be related to life history traits of mouse lemurs, especially seasonal variations in energy balance and fat storage during the resting season [26-29].

In mouse lemurs, glucose and phosphorus levels were higher in the long-day photoperiod. These two parameters are closely related to nutrition or malnutrition in mammals. Despite the fact that blood samples were collected when animals were fasting, high glucose values observed in the summer reflect the increase in food intake during the breeding season. This elevated glucose level during the summer has been previously observed in the black bear, which presented with higher glucose levels during the pre-denning season (July-December) [30]. In the brown hare (*Lepus europaeus*), the highest glucose and phosphorus concentrations were observed in summer and the lowest values were obtained in the fall [31]. As for glucose, higher levels of phosphorus in the summer are related to changes in food intake and to modifications in the regulation of energetic metabolism. Moreover, the relationship between blood glucose and body mass in mouse lemurs in the winter season is consistent with the known relationships between body fat (white adipose tissue) and glucose and food intake, which has been previously shown in mouse lemurs [32].

Additionally, albumin, globulin and total protein levels were significantly higher during the winter season. The significant increase in albumin, globulin and key proteins regulating the colloidal osmotic pressure of blood may be due to the reduction of water consumption and food intake during the winter. Both of these factors have an effect on blood composition and osmolarity by increasing the concentration of certain blood components. Seasonal effects modifying albumin levels have been reported in birds (*Columba livia*), with low values reported in the winter [33]. In mouse lemurs, the marked seasonal rhythm is related to physiological outputs, particularly plasma hormone concentrations of IGF-1 and sexual hormones [19]. Changes in hormone concentrations may lead to significant alterations in protein turnover due to high metabolism in response to differences in photoperiod. However, albumin may consistently vary according to environmental factors such as housing conditions (non-human primates [34]). Moreover, total protein levels may reflect nutritional status as well as kidney and liver functions; low levels of total protein may indicate liver, kidney or absorption disorders. In mouse lemurs, high protein content values during the winter may also be linked to changes in renal function or decreases in muscle mass from less motor activity. High protein content in the winter could also be related to the fact that endogenous lipids are the main source of energy utilized, and proteins are spared during this period [35].

All of the observed seasonal differences in blood biochemistry in mouse lemurs reflect adaptive seasonal changes in energy balance, and these differences do not appear to be clinically significant.

### Age-related differences

Total blood urea nitrogen, globulin and creatinine levels were increased in old animals, independently of gender. These parameters are relevant markers of renal function and are known to decline with age. In mammals, decreased renal function is associated with a drop in glomeruli numbers and decreased renal blood flow and pressure; these changes occur as a result of modified cardiac function or persistent vasoconstriction. In the literature, age-related increases in these markers have been previously observed (BUN, human [15]; GLOB, black bear [30], chimpanzees [11], and humans [15]; CRE, non-human primates [22] and humans [15]).

Age-related alterations in renal function have been observed in mouse lemurs, and most deaths in captivity are due to renal insufficiency [36]. Chronic kidney disease in older animals may explain the age-related increase in creatinine levels.

## Conclusion

Because the Grey Mouse Lemur is considered to be a pertinent model for experimental studies on aging processes [19] and for anti-aging protocols [32,37], this study provides relevant and useful biomarkers to monitor the health status of captive animals. Enzymatic activity markers and electrolytes weakly vary between the sexes and seasons and may be used for assessing the general health status. By contrast, blood parameters linked to renal function or protein metabolism need to be further studied to control for age-related changes in this primate species.

## Methods

### Ethics statement

All experiments were performed in accordance with the Principles of Laboratory Animal Care (National Institutes of Health publication 86–23, revised 1985) and the European Communities Council Directive (86/609/EEC). Studies were conducted based on the agreement of the Internal Review Board at UMR (7179) and in accordance with the recommendations of the Weatherall report (for the use of non-human primates in research). Special attention was paid to the welfare of animals during this work to minimize nociception [38].

### Animals and husbandry

All Grey Mouse Lemurs studied were born in the laboratory breeding colony of Brunoy (Muséum National d’Histoire Naturelle, UMR 7179 CNRS/MNHN, France; Agreement DDPP # D91-114-1) from a stock originating from lemurs caught 45 years ago along the southwestern coast of Madagascar. General conditions of captivity were consistently maintained. Animals were exposed to ambient room temperature (24–26°C) and a relative humidity of 55%. Animals were fed *ad libitum* every two days; fresh fruits (apples and banana) and a homemade mixture of white cheese, ginger bread, baby cereal, milk and egg were provided. Water was always given *ad libitum*.

Biological rhythms of mouse lemurs are dependent on photoperiod. Exposure to long days (>12 h light per day) causes the seasonal activation of reproductive function associated with increased behavioral and physiological activity. In contrast, exposure to short days (<12 h light per day) leads to pronounced fattening, reduced activity, torpor and complete sexual rest in both sexes [39]. In the breeding colony, seasonal variations of physiological functions are artificially maintained by alternating a 6-month period of summer-like long-days (LD: 14 h of light / day) with a 6-month period of winter-like short-days (SD: 10 h of light / day) under artificial light. Artificial light was provided by fluorescent tubes (white light, 250 lux, wavelength peak at 488 nm). Seventy-eight animals were used in the study: 43 females (aged 1 to 10 years) and 35 males (aged 1 to 8 years). None of the females were pregnant or lactating. To ensure complete stabilization of the physiological status in animals following changes in photoperiod, mouse lemurs were studied 1–2 months after the onset of the LD photoperiod (breeding season) and 1–2 months after the onset of the SD photoperiod (resting season). All tests were conducted during the 4 hours before the nocturnal active period.

The median survival time, the time at which half of the population has died, was generally used to delineate the age groups within a population. In the captive colony, the median survival time of mouse lemurs is 4.9 years for females and 5.7 years for males [19]. Consequently, three age groups were used: young animals (1–3 years old), middle-aged animals (4–5 years old) and old animals (6–10 years old).

### Total blood sample collection

Blood collections were taken via the saphenous vein without anesthesia during the daily resting phase just before the nocturnal activity period. Therefore, animals were in a fasting state. Blood samples (100 to 150 μL, 0.1% of total blood volume) were collected in lithium-heparinized tubes just before analysis. The body mass of each animal was measured before each measurement to monitor body mass variations.

### Measurements of biochemical levels

Measurements of blood components were made using a VetScan VS2 Chemistry Analyzer (© 2002, Abaxis, Inc., Union City, CA, United States), following the manufacturer’s instructions. This system uses dry and liquid reagents to provide veterinary *in vitro* quantitative determinations of specific physiologic parameters. The VetScan Comprehensive Diagnostic Profile (Comprehensive Diagnostic Profile, Abaxis, Union City, CA) was used in the study and provided values for the following biochemical markers in lithium-heparinized whole blood: total protein concentration (TP), albumin concentration (ALB), total bilirubin concentration (TBIL), globulin concentration (GLOB), blood urea nitrogen (BUN), glucose concentration (GLU), creatinine (CRE), inorganic phosphorus (PHOSP), sodium (Na), potassium (K), calcium (Ca), alanine aminotransferase activity (ALT), amylase (AMY) and alkaline phosphatase activity (ALP).

Whole blood samples obtained by venipuncture were homogenized by gently inverting the collection tubes several times prior to transferring to the rotor. The VetScan Chemistry Analyzer automatically calculates and prints the analyte concentrations in the sample. Physical interferents, including blood hemolysis (HEM), icterus (ICT) and lipemia (LIP) may alter the component concentrations. The VetScan Chemistry Analyzer suppressed any results that were affected by >10% interference from HEM, LIP or ICT. In our samples, the mean value of these parameters ranged from 0 to 3%, and there were no significant differences between seasons, sexes and ages (p>0.1).

### Coefficient variation and out of range individuals

Coefficient variations (CV%) were determined on the basis of the results of a pilot study, which was conducted prior to this study. For the pilot study, three females from the breeding colony (2–3 years old) were used. Because it is difficult to collect a high number or high volume of blood from the Grey Mouse Lemurs, we collected 200 μL of total blood from each female and used two distinct VetScan Comprehensive Diagnostic Profiles per sample. For these three mouse lemurs, the coefficient of variation averaged 10% (individual values: 9.04%, 11.78% and 8.95%, respectively). These values are in agreement with the intra-test CV% provided in the manufacturer’s instruction manual for other mammal species.

For some biochemical parameters, the VetScan analyzer reported out-of-range values; these mainly occurred in male mouse lemurs exposed to the long photoperiod. Creatinine levels in two males were too low for detection by VetScan. Similarly, the sodium level was below 100 mmol/L in one male and could not be measured by VetScan. For albumin measures, four males exposed to the long photoperiod had a rate below the limit of detection (1.5 g/dL). Finally, three females exposed to the long-day season had levels of potassium greater than 8.5 mmol/L, beyond the detection limit of the auto-analyzer. For some individuals, measurements were not obtained for some criteria because the blood sample was hemolyzed during the assay.

### Data analysis and statistics

The blood biochemical parameters were evaluated separately according to season, sex and age. Using the Shapiro-Wilk goodness-of-fit test, most variables were not normally distributed, and they were log transformed for analysis. The median, 25% and 75% quartiles, and range (max-min) were determined for males and females (Tables 1 and 2). Three-way ANOVA and post-hoc tests were used to assess the potential effects of sex, season and age. For each biochemical marker, crossed effects: sex*season, sex*age, season*age and sex*season*age were also analyzed. Simple linear regressions were used to determine the significance of correlations between blood variables and body weight or age. All analyses were performed with R version 2.12.0 (R Development Core Team, Vienna, Austria). A p-value of < 0.05 was considered to be significant, and all values are expressed as the means ± standard errors of the mean (SEM).

## Competing interests

The authors have no non-financial competing interests to declare. The study was funded by UMR CNRS/MNHN 7179. The funders had no role in study design, data collection and analysis, decision to publish, or preparation of the manuscript. The authors declare that the research was conducted in the absence of any commercial or financial relationships that could be construed as a potential conflict of interest.

## Authors’ contribution

JM, OD and LH conducted the experiments. FA and MP conceived the study and its design. JM performed the statistical analysis. JM, MP, FA and OD were involved in drafting the manuscript and critically revising it for important intellectual content. All authors have given final approval of the version to be published.
